# Enhancing Diversity and Productivity of the HIV Behavioral Research Workforce through Research Education Mentoring Programs

**DOI:** 10.1007/s10461-019-02520-w

**Published:** 2019-05-25

**Authors:** David M. Stoff

**Affiliations:** National Institute of Mental Health, 5601 Fishers La, Bethesda, MD 20892 USA

**Keywords:** HIV/AIDS, Diversity, Mentoring, Training

## Abstract

Mentoring programs to enhance diversity in the HIV research workforce, using the research education grant mechanism (R25), were addressed to promote new investigator development in HIV-related behavior and social sciences. The utility and benefits of the R25 mechanism were discussed. Outcome data from publication history and funded grants of mentees from the major racial and ethnic minority backgrounds indicated the success of these programs in promoting HIV-related career development. Next steps and future challenges were addressed for further enhancing the HIV research workforce.

## Introduction

Recently, there has been recognition of a compelling need for improved strategies to promote greater diversity in the biomedical, clinical, and behavioral research workforce and to eliminate disparities [1]. These strategies include formalized mentoring programs, which are known to offer benefits to the recruitment, training, and retention of diverse trainees and faculty (e.g., [2]). The NIH provides leadership in enhancing the diversity of the scientific workforce, in training the next generation of scientists, and in developing research capacities throughout the country. One of the major vehicles to accomplish this is the NIH Research Education Grant (i.e., R25 programs). The Division of AIDS Research of the National Institute of Mental Health (NIMH) employs the R25 to provide funds to institutions for developing and implementing education programs that disseminate scientific discoveries within a thematic research area, promote appreciation and interest in mental health research, offer hands-on exposure to research, and develop specific research skills in needed areas. The utility of the R25 to the science of public health lies within its multiple goals (i.e., training of workforce, enhancing diversity, facilitating recruitment, fostering improved research understanding) and multiple platforms (i.e., mentoring activities, research experiences, courses for skills development, curriculum or methods development, outreach). Moreover, the R25 is unique in its national reach in the research education of participants, in-depth focus on a specialized research content, and provision of personnel support to carry out its aims.

One pressing issue facing today’s research pipeline is the underrepresentation of individuals from diverse racial and ethnic backgrounds [3]. Mentorship is one of the most frequently cited components of a successful research career [2] and is an important way to remediate diversity related barriers in the field [4]. For example, the Diversity Program Consortium [5] is a trans-NIH program that has a strong mentorship component to enhance the participation and persistence of individuals from underrepresented backgrounds in biomedical research careers. However, there is little attention to behavioral research issues. In view of the landmark and paradigmatic advances that have been made in HIV prevention and treatment [6], the Division of AIDS Research (DAR) of NIMH focused on HIV-related behavioral and social sciences (using R25 grants for research mentoring programs) to build a pipeline and to achieve rapid growth of a diverse HIV research workforce.

Past work addressed the conceptual background and principles of the HIV research mentoring process for strengthening racial and ethnic diversity in the research workforce [7]. This paper reviews the most salient features and some outcome data from the NIMH/DAR portfolio of R25 programs. The over-arching goals of these R25 programs are to: (i) complement and/or enhance the training of a workforce to meet the nation’s biomedical, behavioral and clinical research needs (see ACD Working Group on Biomedical Workforce [8]; and (ii) enhance the diversity of the biomedical, behavioral and clinical research (see Notice of NIH’s Interest in Diversity) [9]. In addition to R25-based programs, there have been other research programs to develop minority HIV investigators. This includes the Minority HIV/AIDS Research Initiative (MARI) for junior minority HIV scientists in health disparities [10]; HIV Prevention Trial Network, HPTN, minority scholars program for postdocs and junior M.D. researchers [11]; and HIV Vaccine Trial Network, HVTN, minority scholars program for medical student researchers [12]. One of the largest diversity mentoring initiatives is the NIH-supported National Research Mentoring Network which employs structured mentorship and networking experiences to enhance the training and career development of individuals from diverse backgrounds who are pursuing biomedical and behavioral research careers [13].

Efforts to diversify the workforce are grounded in the notion that increasing workforce diversity will lead to decreased health disparities and health equity among racial/ethnic minority populations. Meeting the clinical and research needs of an increasingly diverse U.S. population is an issue of national concern, yet, data indicate that we are not currently benefitting from the talents and contributions of people who represent the diversity of all citizens (e.g., [14]). Moreover, going beyond the impact of diversity on disparities reduction, Page [15] discusses how diversity leads to superior outcomes, citing research showing that diverse teams working together and capitalizing on innovative ideas and distinct perspectives out-perform homogenous teams. Scientists and trainees from diverse backgrounds and life experiences bring different perspectives, creativity, and individual enterprise to address complex scientific problems.

In recent years, a substantial body of literature has explored the extent to which diversity in the composition of the health-care workforce may be used as a tool to enhance interactions and, therefore, reduce disparities in health and health care in the United States (e.g., [16, 17]). NIH has played a leading role in this regard. The Advisory Committee to the NIH Director’s (ACD) Working group on Diversity in the Biomedical Research Workforce [3] deliberated on ways in which enhanced diversity might lead to reduction and eventual elimination of the reported African American/Black funding disparities [18]. Besides disparities reduction, there are many other benefits that flow from a diverse NIH-supported scientific workforce, including: fostering scientific innovation, enhancing global competitiveness, contributing to robust learning environments, improving the quality of the research, advancing the likelihood that underserved or health disparity populations participate in and benefit from health research, and enhancing public trust. Nevertheless, despite the multiple benefits of diversity and the long-standing efforts of NIH (and other agencies), the biomedical research workforce does not reflect the diversity of the overall population. For example, in a recent article [19] it is estimated that only 4% of the nation’s scientific research faculty are African American, 4% are Hispanic, 0.2% are Native American, and 0.1% are Hawaiian/Pacific Islander. In addition to significant underrepresentation in the sciences for the major racial/ethnic minority groups, significant losses are observed at every stage in the training process [20]. Thus, independent research investigators from these groups are rare.

## General Methods: Diversity Research Education Programs

From 2006 to 2017, NIMH/Division of AIDS Research, received 98 new and renewal applications (36% success rate) in response to a series of five sequentially published funding opportunity announcements (data obtained from NIH/ERA QVR database—Query, View, and Report, a tool that provides detailed information about grant applications and awards). Initially, this initiative was designed exclusively for mentee participants who are underrepresented in science and engineering [14] and was expanded to include all mentee participants (from funded R25s) but a focus on participants from underrepresented racial and ethnic backgrounds was retained.

Mentees from three underrepresented, underserved populations (i.e., African Americans, Latinos, Native Americans) were selected for each of the R25 programs. These diverse mentees were at multiple career levels (i.e., predoctoral, postdoctoral, or early career), with the greater majority at the postdoctoral or early career levels. Mentee home institutions were across the nation, but the majority of R25 programs were from the East Coast and West Coast. The criteria for mentee recruitment/selection, mentor–mentee matching procedure, and program evaluation were specific to the individual R25 programs.

Mentees experienced two major activities that were structured around two central themes, community engagement ***(***the process of working collaboratively with groups of people) and transdisciplinary team science (disciplinary integration on methodologic and theoretic aspects): (i) *Mentoring and career development* by senior faculty, established researchers, and experienced mentors that included technical expertise, career advice, insight, and professional skills development to cohorts of culturally skilled scientists who were underrepresented in the biomedical sciences, to increase their potential to become competitive grantees. Mentoring activities included instruction in presentations skills, scholarly writing, and grantsmanship; promoting successful transitions from one career stage to another; providing opportunities for the development of leadership skills, and HIV topic-related didactics/courses. As indicated in Table 1, the major mentoring delivery strategy was team mentoring but other strategies were also used (e.g., one-on-one, peer, online). Some programs incorporated mentor training protocols for improved mentoring skills and more standardized mentoring. (ii) *Direct (i.e., hands*-*on) research experiences* in HIV thematic research areas, as noted in Table 1; the greater majority were in HIV-related behavioral and social sciences and only a few in NeuroHIV. These research experiences were pilot research projects with the goal of helping mentees obtain preliminary results which could be leveraged for larger grant applications, Thus, pilot funds provided hands-on exposure to research to enhance their experiences, research skills, and knowledge base relative to NIMH/DAR scientific mission areas and cross cutting conceptual and methodologic approaches. Pilot research projects were approved both by the corresponding R25 program and NIMH program staff, in accordance with Priorities and Guidelines for NIH OAR Research [21]. The various R25 programs in HIV-related behavioral and social sciences fall within seven different HIV content areas and the majority of R25 grants were within HIV prevention or HIV care continuum (Table 1). The fastest growing HIV content area was HIV implementation science, consistent with its growth in public health [22] and HIV [23].

**Table 1:** HIV R25 Programs supported by the National Institues of Health

R25 Institution (P.I.)	Project title	Abstract link	Program website	Career level targeted	Project period	No. mentees enrolled/graduated past five yrs (2013–2017)	Main activities	HIV research focus
American Psychological Association (Tiffany Towsend and Velma Murry)	Cyber mentors: A training program for HIV/AIDS researchers working on health disparities	Abs	http://www.apa.org/pi/aids/programs/cyber/	Postdoctoral, Early Career (all from diverse racial/ethnic groups)	2011–2015	33/30	Pilot research; online mentoring	Prevention
Brown University (Amy Nunn and Timothy Flanigan)	The brown initiative in HIV and AIDS clinical research for minority communities	Abs	https://brownr25fellowship.wordpress.com/	Postdoctoral (all from diverse racial/ethnic groups)	2008	42/36	Pilot research; Team, one-on -one mentoring	Community-based and implementation science
Columbia University (Nabila El-Bassel and Elwin Wu)	HIV Intervention Science Training Program for Promising New Investigators from Underrepresented Groups (HISTP)	Abs	https://histp.columbia.edu/	Postdoctoral (all from diverse racial/ethnic groups)	2007	12/11	Pilot research; Team, one- on-one, online mentoring	Implementation science, bio-behavioral intervention research
Johns Hopkins University (Amanda Brown)	Project pipeline Baltimore: A brain sciences program for high school students	Abs	https://www.hopkinsmedicine.org/neurology_neurosurgery/research/labs/jhu_nimh/jhibs/	High school students (all from diverse racial/ethnic groups)	2013–2018	57/57	Shadowing research; Team, one-on-one mentoring	Neuroscience education
Johns Hopkins University (Justin McArthur)	Translational Research in Neuro-AIDS and Mental Health	Abs	https://www.hopkinsmedicine.org/neurology_neurosurgery/education/neurology-electives/neuro-aids-research-course/index.htm	Postdoctoral Predoctoral (majority from diverse racial/ethnic groups)	2007	34/28	Pilot research; Team, one-on-one mentoring	Neuropathogenesis, neuroprotection, and therapeutics
Icahn School of Medicine at Mount Sinai (Susan Morgello)	The Mount Sinai Institute for NeuroAIDS Disparities	Abs	http://www.msinad.org/	Postdoctoral Predoctoral (all from diverse racial/ethnic groups)	2007–2017	16/16	Pilot research; Team, one-on-one, mentoring	NeuroHIV, neuropsychology, neurology, and neuropathology
San Francisco Department of Public Health (Fuchs/McFarland)	Summer HIV/AIDS research program	Abs	http://www.sharpinternship.org	Undergraduates (all from diverse racial./ethnic groups)	2012–2017	24/24	Shadowing; pilot research, Team, one-on-one, mentoring	Biomedical/behavioral prevention, implementation science, disparities
University of California, San Francisco (George Rutherford)	International Traineeships in AIDS Prevention Studies (ITAPS)	Abs	http://globalhealthsciences.ucsf.edu/international-traineeships-aids-prevention-studies-itaps	Postdoctoral Early career (all from low and middle income countries)	2002	70/70	Mentor training; scientific writing and grant writing	Psychosocial prevention and implementation science
University of California, Los Angeles (Jesse Clark)	South American Prevention of HIV Research Training Program (SAPHIR)	Abs	https://www.uclasaphir.com/	Predoctoral; Postdoctoral (majority from diverse racial/ethnic groups)	2010	21/21	Pilot research; Team, one-on-one mentoring	Global prevention (includes epidemiology, behavioral prevention, biomedical prevention, therapeutics)
University of Puerto Rico (Carmen Zorrilla)	University of Puerto Rico Mentoring Institute for HIV and Mental Health Research: Developmental, competency-based Mentoring for Junior Investigators	Abs	http://mentoringinstituteforhivandmentalhealth.weebly.com	Predoctoral, early career; (all from diverse racial./ethnic groups)	2008	15/10	Pilot research; Team, one-on one, and peer mentoring	Women’s mental health; Stigma, Violence, adherence; clinical and Behavioral prevention
University of California San Diego (Dilip Jeste)	Sustained Training in Aging and HIV Research (STAHR) Program	Abs	https://medschool.ucsd.edu/som/psychiatry/research/stahr/Pages/default.aspx	Postdoctoral Early career (mixed racial/ethnic groups)	2016	6/0	Group research project; Team, on line mentoring	Aging and mental health
University of California, San Diego (Mariana Cherner)	Interdisciplinary Research Fellowship in NeuroAIDS	Abs	https://hnrp.hivresearch.ucsd.edu/index.php/irfn-home	Postdoctoral	2007	24/20	Research part of ongoing project; One on one and team mentoring	Clinical and basic neuroscience
University of California, San Francisco (Torsten Nielands)	Training for Scientists Conducting Research to Reduce HIV/AIDS Health Disparities	Abs	https://prevention.ucsf.edu/education/vp/	Postdoctoral, Early career (all from diverse racial./ethnic backgrounds)	2003	9/6	Pilot research; Team, one-on-one, online, and peer mentoring	Prevention and treatment
University of Puerto Rico (Carmen Zorrilla)	University of Puerto Rico Mentoring Institute for HIV and Mental Health Research: Developmental, competency-based Mentoring for Junior Investigators	Abs	http://mentoringinstituteforhivandmentalhealth.weebly.com	Early career, Predoctoral (all from diverse racial./ethnic backgrounds)	2008	15/10	Pilot research; Team, one-on one, and peer mentoring	Women’s mental health; Stigma, Violence, adherence
University of Washington (Karina Walters)	Indigenous HIV/AIDS Research Training Program	Abs	http://iwri.org/research/ihart-2/	Early career (all from diverse racial./ethnic backgrounds)	2009	17/9	Pilot research; team mentoring	Psychosocial prevention
Yale University (Trace Kershaw, Barbara Guthrie)	Research Education for Diverse Scholars (REIDS)	Abs	http://cira.yale.edu/training/reids/research-education-institute-diverse-scholars-reids	Early career (all from diverse racial./ethnic backgrounds)	2010	24/20	Pilot research; one-on-one and peer mentoring	Community based implementation science

## Outcomes of R25 HIV Research Programs

Publications and external funding are considered the most commonly used definitions of academic success. To assess outcomes after R25 completion (i.e., publication history, NIH awarded grants), a subset of mentees was selected from HIV R25 programs with (a) exclusive focus on diversity and (b) pilot research projects as research experiences. For publication data, 40 mentees (approximately 4–6 mentees from each of seven R25 programs) were selected and assessed according to Medline database described below. For grant data, 30 mentees (approximately five mentees from each of six R25 programs) were selected, based on the official input of mentee/trainee appointment data from X-Train into the NIH computer system, to assess publication history and funded grants. To allow time for publications and grants to appear after R25 completion, publications and grants were examined 2–5 calendar years after R25 completion.

### Publication History

Results showed that 25% of the mentees published more than ten articles, 18% published between six and ten articles, and 48% published one to five articles (see Table 2). These data indicate considerable variation in publication productivity that is due primarily to extremely high or extremely low publication numbers. In addition, the mentee was the lead author on 31% of the articles and 83% were multi-authored. This indicates that a reasonably large portion of mentees assumed leadership role in publications and there was an extremely high degree of collaboration, often with other mentees from the same R25 program (data not shown). These data suggest that subsequent studies of publication history might employ methods of social network analyses to characterize collaboration patterns among mentees. When HIV publications were separated according to whether they were directly related to HIV (i.e., those that mention HIV/relevant behaviors in the title or in the abstract and provide knowledge about HIV/relevant behaviors) or indirectly related to HIV (i.e., those that do not mention HIV/behavior in the title or in the abstract, yet can inform HIV research), analyses indicated slightly more HIV-indirectly related publications (58%) than HIV-directly related publications (42%). This suggests that R25 programs varied in the level of HIV research with some studying either HIV or pre-HIV foundational research, to the extent that directly-related and indirectly-related HIV publications reflect this varied emphasis.

**Table 2:** Outcomes of subset of R25 mentees

Publications (N = 40 mentees)	N (% mentees)
No articles	4 (10%)
1–5 articles	19 (48%)
6–10 articles	7 (18%)
More than 10 articles	10 (25%)
Publications (N = 271 articles)^a^	N (% articles)
Mentees’ lead author articles	85 (31%)
Mentees’ multi-authored articles	225 (83%)
Research Grants (mentee as P.I.) (N = 30 mentees)^b^	N (% mentees)
R01	5 (17%)
R03/R21/R34	4 (13%)
Other Grants (i.e., R15, R56, U01)	5 (17%)
Total RPGs	14 (47%)
Training Grants (mentee as P.I.) (N = 30)^b,c^	N (% mentees)
Career awards	7 (23%)
NRSA Ts, Fs	4 (13%)
LRP	14 (47%)
Total training awards	25 (83%)

Publication history: To measure publication productivity for each individual, the number of publications in each calendar year, two to five years after R25 completion, was recorded from the National Library of Medicine’s MEDLINE, according to modification of the method described by Mason et al. [24]. The Medline database was accessed (around Jan 2018) for each of the mentees. Publications were counted within each of the following categories by searching in the title or in the abstract: [1] directly-related HIV/behavioral publications (i.e., “HIV” appeared and behavior was described; [2] indirectly related HIV/behavioral publications (i.e., “HIV” did not appear but issues that might inform HIV-behavioral research did appear, such as risky behavior, minorities, nonHIV infectious diseases or assessment/methodology; [3] first-authored publications (i.e., mentee was first author); [4] collaborative publications (i.e., mentee was associated with at least two other co-authors)

NIH Research Grant Funding: Grant data was collected using the iSearch tool created by the NIH Office of Portfolio Analysis, which links unique person IDs to NIH grants

^a^Articles directly and indirectly related to HIV/behavior

^b^Not mutually exclusive category

^c^Training awards may have occurred before or after R25

### NIH Research Grant Funding

As shown in Table 2, 47% were awarded NIH RPGs (Research Project Grants) during the time period of 2–5 years after R25 completion. Almost one-third of mentees (i.e., 30%) received R01 s or R03 s or R21 s/R34 s Subsequent to receipt of these R-series grants, about one-quarter of mentees (i.e., 23%) were awarded a Career Development “K”award, and were, therefore, on the trajectory to research independence. However, this can not exclusively be attributed to the R25 experience since the R25 was only one contributor to their multiple research training experiences. About one-half of the mentees also were awarded a NRSA T or F (i.e., 13%) and LRP, loan repayment program (47%). LRPs not only strengthen retention in the research pipeline but are also formative training type awards, based on an applicant’s potential to build and sustain a research career.

Given the absence of a non-R25 comparison group, these correlative outcome data are limited in determining the direct impact of R25 research education on publication and grant outcomes. Nevertheless, conclusions from these outcome data suggests that R25s may play an important role in further engaging and promoting relevant career outcomes. Moreover, future evaluative research in this area should employ logic models, i.e., theoretically and empirically based frameworks that allow for positing research questions about program effectiveness, such as what is the relationship of the program components to participant outcomes (e.g., [25]). A major benefit of such a theoretically guided approach is the ability to operationalize short (e.g., obtain HIV grant support), intermediate (e.g., demonstrate leadership in HIV field), and long-term (e.g., strengthen HIV research workforce) outcomes that result from meeting the core competencies (e.g., research excellence, collaboration, scientific writing) that constitute the components of the mentoring program.

### Concluding Remarks

A portfolio of HIV research education programs for mentoring and research experiences, targeting emerging investigators from racial and ethnic minorities, has been described. The effectiveness of these programs was indicated by outcome data on publications and NIH grants. However, it is unlikely that the R25 program, in itself, leads to career success in the field of HIV/diversity/behavior, although it may be one of multiple contributors. The presented data are only a partial description from these R25 programs. Other features are program specific and were not presented (e.g., prospective evaluations and longer term outcomes; mentee satisfaction; effects of mentor training). The most general conclusion that can be offered is that the R25 program may play an important role to welcome, engage, mentor, and promote the career of emerging HIV investigators.

Looking ahead to “next steps,” it could be beneficial to further enrich some of the most important features and components of the current R25 mentoring programs. These next steps could potentially include: (i) extension and broadening of pipeline to include early academic/career development and trainees from a variety of academic backgrounds; (ii) improved recruitment through community-based participatory research and social networking approaches; (iii) establishing mentee proficiency in interdisciplinary competencies and overcoming challenges of interdisciplinary mentorship; and (iv) inter-institutional/consortia partnerships to further ensure collaborations for a research network.

Some challenges and opportunities for diversity-focussed training programs have been identified by others [19]. The following data-driven challenges are relevant to future R25s or other similar programs.

#### Challenge 1: Workforce Dynamics

Understand the dynamics that produce successful scientists, the characteristics and factors that make investigators successful, including diversity among subgroups and how people make career decisions. What factors influence emerging scientists from diverse backgrounds to enter, exit, and sustain a behavioral and biomedical research career? How do minority mentees develop the requisite competencies at different training and career stages? These relevant factors and variables of career success might be analyzed through regression models.

#### Challenge 2: “Leaky” Pipeline/Retention

Not all individuals flow equally through the pipeline; rather, race, ethnicity and gender substantially influence the forward momentum through the pipeline at succeeding stages. Racial, ethnic, and sexual minorities are more likely to “leak” out of the pipeline, resulting in low numbers of minorities at every stage of career development [20]. Thus, the “leaky” pipeline appears to be an issue of retention, i.e., attrition loss. This suggests that targeting retention at successive career stages (longitudinally) (e.g., baccalaureate to predoctoral, postdoc to K, K to R01) could mitigate some of this attrition. Targeted programs can be developed to facilitate research career development and retention by identifying those characteristics that best predict persistence and retention in retention careers (such as self-efficacy).

#### Challenge 3: Integration of R25

Free-standing R25 mentoring program may be more effective when partnered with other existing, effective programs. Such partnering might involve embedding an R25 program within HIV clinical trial networks (e.g., HPTN) or within cohort projects (e.g., MACS or WIHS). Program integration may enable leveraging of existing resources to develop programs with more immediate impact, utilizing ongoing databases for preliminary mentee projects and access to senior, experienced mentors. Such integration promotes translational and transdisciplinary approaches and can lead to increased understanding of health disparities [26]. Additionally, an NIH Advisory Group to the Director [17] recommended that partnering would help to implement a system of mentorship networks for underrepresented minority students that will provide career guidance throughout their career development.

#### Challenge 4: Institutional Mentoring Climate

Sociocultural factors that affect recruitment and retention are critical regarding diversity workforce considerations for R25 programs and other relevant educational and training career development programs. Among the most important individual and institutional-based sociocultural factors are unconscious bias, stereotype threat, benevolent racism/sexism, microaggressions, belonging, cultural climate, and identity. For example, multiple studies have shown that implicit or unconscious bias (i.e., attitudes or stereotypes that affect our understanding, judgment, actions, and decisions) may contribute to health disparities and lead to negative health outcomes [27]. It has been suggested that implicit bias and other sociocultural obstacles interfere with the development of the infectious disease workforce [28]. Increased attention is necessary to institutional factors that “warm” the mentoring climate and aid in destigmatizing career development challenges for individuals from diverse racial and ethnic backgrounds.

Addressing these challenges will provide important information on the nature of mentoring interventions that may be successful in promoting the diversity and stability of the workforce. An understanding of dynamics may allow us to propose the development of asset models and leadership opportunities, rather than solely deficit models and remediation. By combining institutional policies that promote a research culture and climate for diversity with quality, evidence-based programs and networks of support, we can go beyond an individual skill-based approach to generate a sustainable research workforce that will inform policy. At an institutional level, a culture of mentoring needs to be fostered as a revered value. Finally, mentoring for diversity, alone, is not enough. A comprehensive approach to education and training of new emerging scientists is needed where mentoring operates together with other factors, such as infrastructure development, diversity inclusion/cultural competency, academic leadership and research management, experiential learning, and a contemporary curriculum.
